# Mediators Linking Childhood Adversities and Trauma to Suicidality in Individuals at Risk for Psychosis

**DOI:** 10.3389/fpsyt.2017.00242

**Published:** 2017-11-20

**Authors:** Stefanie J. Schmidt, Frauke Schultze-Lutter, Sarah Bendall, Nicola Groth, Chantal Michel, Nadja Inderbitzin, Benno G. Schimmelmann, Daniela Hubl, Barnaby Nelson

**Affiliations:** ^1^University Hospital of Child and Adolescent Psychiatry and Psychotherapy, University of Bern, Bern, Switzerland; ^2^Department of Psychiatry and Psychotherapy, University of Cologne, Cologne, Germany; ^3^Department of Psychiatry and Psychotherapy, Medical Faculty, Heinrich-Heine University, Düsseldorf, Germany; ^4^Orygen, The National Centre of Excellence in Youth Mental Health, University of Melbourne, Melbourne, VIC, Australia; ^5^Centre for Youth Mental Health, University of Melbourne, Melbourne, VIC, Australia; ^6^Developmental Clinical Psychology Research Unit, Faculty of Psychology and Educational Sciences, University of Geneva, Geneva, Switzerland; ^7^University Hospital of Child and Adolescent Psychiatry, University Hospital Hamburg Eppendorf, Hamburg, Germany; ^8^University Hospital of Psychiatry and Psychotherapy, University of Bern, Bern, Switzerland

**Keywords:** psychosis, mediation, depression, suicidality, basic symptoms, attenuated psychotic symptoms

## Abstract

Suicidality is highly prevalent in patients at clinical high risk (CHR) for psychosis. Childhood adversities and trauma are generally predictive of suicidality. However, the differential effects of adversity/trauma-domains and CHR-criteria, i.e., ultra-high risk and basic symptom criteria, on suicidality remain unclear. Furthermore, the underlying mechanisms and, thus, worthwhile targets for suicide-prevention are still poorly understood. Therefore, structural equation modeling was used to test theory-driven models in 73 CHR-patients. Mediators were psychological variables, i.e., beliefs about one’s own competencies as well as the controllability of events and coping styles. In addition, symptomatic variables (depressiveness, basic symptoms, attenuated psychotic symptoms) were hypothesized to mediate the effect of psychological mediators on suicidality as the final outcome variable. Results showed two independent pathways. In the first pathway, emotional and sexual but not physical adversity/trauma was associated with suicidality, which was mediated by dysfunctional competence/control beliefs, a lack of positive coping-strategies and depressiveness. In the second pathway, cognitive basic symptoms but not attenuated psychotic symptoms mediated the relationship between trauma/adversity and suicidality. CHR-patients are, thus, particularly prone to suicidality if adversity/trauma is followed by the development of depressiveness. Regarding the second pathway, this is the first study showing that adversity/trauma led to suicidality through an increased risk for psychosis as indicated by cognitive basic symptoms. As insight is generally associated with suicidality, this may explain why self-experienced basic symptoms increase the risk for it. Consequently, these mediators should be monitored regularly and targeted by integrated interventions as early as possible to enhance resilience against suicidality.

## Introduction

Suicide is defined as the deliberate act to take one’s own life. With over 800,000 persons having completed suicide worldwide in 2012 (1), suicide is among the top 20 causes for mortality in the world. Notably, suicide rates in adolescents have increased in recent years, making suicide the second leading cause of death globally in individuals aged between 15 and 29 years (1). Non-lethal suicidality, including suicidal ideation (i.e., thinking about killing oneself) and suicide plans as well as attempts, is even more prevalent and substantially increases the risk of death by suicide (2). Among psychiatric patients, risk of suicidality is generally increased, in particular in patients with psychotic disorders (3). In psychosis, it is highest in the early stages of the disorder (4). Accordingly, the first meta-analysis of clinical high risk (CHR)-patients including 21 studies with 2,808 participants revealed high prevalence rates of 66.1% for current suicidal ideation and 17.7% for lifetime suicide attempts (5).

In both general population and psychiatric samples, childhood adversities and trauma are one of the main psychological predictors of suicidality (6–8). While suicidality seems to be related to childhood adversities and trauma in patients with first-episode psychosis (9, 10), this link has not yet been studied sufficiently in CHR-patients. Furthermore, most previous studies in general population, patient and in particular in CHR-samples have investigated potential predictors of suicidality in isolation without analyzing their interplay and their relative contributions to suicidality simultaneously. Consequently, we still have a limited understanding of the mechanisms linking adversities and trauma to suicidality (7). Therefore, based on the current literature, this study aimed to test theory-based models about potential mechanisms contributing to the relationship between childhood adversities/trauma and suicidality in a sample of CHR-patients. A CHR-state of psychoses was alternatively defined by the ultra-high risk (11) and the basic symptom criteria, including cognitive disturbances (COGDIS) and cognitive-perceptive basic symptoms (COPER) (12).

Experiences of adversities and trauma are highly prevalent in CHR-patients (13, 14). They are related to the development of psychopathology, including depressive and psychotic symptoms, which function as precipitants of suicidality (14–17). Models to explain this relationship in CHR-patients include stress-vulnerability and stress-sensitization models (14). They postulate that exposure to trauma as a major stressor interacts with an individual’s vulnerability. This interaction leads to a dysregulation of the stress-response system and an increase in the susceptibility to develop psychopathology, such as psychotic symptoms. After an experience of first psychotic symptoms, the stress-threshold is lowered for the development of even more severe psychopathology (13, 18). Furthermore, the hopelessness theory of suicidality (19, 20) posits that early adversity can facilitate the development of a negative cognitive style as an enduring vulnerability factor characterized by external control beliefs (i.e., events are mainly controlled by others and outside of personal control) as well as negative self-evaluations (e.g., being worthless, lack of self-efficacy). Such a cognitive style has shown to trigger threat anticipation, paranoid ideas, depressive symptoms, and finally suicidal ideation (21, 22). The interpersonal theory of suicidality (20, 23, 24) suggests that the experience of adversities/trauma increases the risk for suicidality through thwarted belongingness and perceived burdensomeness, which are especially pronounced in patients with psychosis due to diminished social connectedness as well as stigma (20, 25) and experiences of being a burden on caregivers (26). In line with these current models of suicidality, childhood adversities and trauma were associated with poor emotion-focused coping, more distress, negative self-beliefs, and depressiveness in CHR-patients (27, 28). Suicidality was significantly related to poor self-esteem (29) and high levels of distress as well as depressiveness (30). However, all of these studies in CHR-patients have not yet integrated these potential mediator variables within one model. Furthermore, while studies demonstrated that a CHR-status, in particular defined by attenuated psychotic symptoms, was linked to childhood adversities as well as trauma and suicidality (30–32), basic symptom criteria have not yet been investigated for their potential association with suicidality.

Against this background, we hypothesized the following mechanisms: (1) childhood adversities and trauma are significantly associated with suicidality and (2) this relationship is mediated by psychological variables: dysfunctional coping and competence/control belief pattern. With regard to the second mechanism, it is noteworthy that social-learning theory and empirical results posit that having positive beliefs about one’s own competencies (i.e., high self-efficacy) and about internal, personal controllability over events are associated with the use of more positive and less negative coping-strategies (33–35). However, some studies have also found the reverse sequence, i.e., positive coping-strategies being associated with high levels of positive competence-beliefs and perceived internal control (36, 37). Therefore, we examined both directions of the second assumed psychological mediators in alternative models. Furthermore, we hypothesized (3) that the mediation effect of psychological variables on suicidality is mediated through increased symptom levels [brief limited intermittent psychotic symptoms (BLIPSs), attenuated psychotic symptoms, COPER/COGDIS, and depressiveness]. Furthermore, potentially confounding variables (age, gender, educational level, current comorbid axis-I disorders) (5, 38) were included as covariates directly influencing suicidality.

## Materials and Methods

### Sample

Clinical high risk-patients were aged between 8 and 40 years as this age-range is associated with the highest probability of psychotic development across gender (39). They were recruited from consecutive referrals to the Early Recognition and Intervention Center for mental crisis (FETZ) Bern between December 2010 and May 2016. Participants had to meet any ultra-high risk or basic symptom criterion. They were excluded if they had a medical, neurological, or substance use disorder accounting for their mental problems. To ensure excellent data quality, diagnostic assessments were performed by trained psychologists, who received weekly supervision. All participants provided written informed consent and parental consent, if they were under the age of 18. The ethics committee of the University of Bern approved the study.

### Instruments

To avoid an age-bias, we administered the same tool when the respective instrument was validated for its application in adults as well as children/adolescents. When results of validation-studies suggested age-differences, we used well-validated children/adolescent- and adult-versions of the same instrument {i.e., Schizophrenia Proneness Instrument [SPI-A/SPI-CY (40, 41)]; German Stress-Coping-Questionnaires [SVF-120/SVF-KJ (42, 43)]; Mini International Neuropsychiatric Interview [MINI/MINI-KID (44, 45)]} or applied the age-adapted test norms available for the same instrument, i.e., German Competence and Control Beliefs Questionnaire [FKK (46)].

#### CHR for Psychosis

The Structured Interview for Psychosis-Risk Syndromes [SIPS (47)] was used to evaluate the presence of the ultra-high-risk criteria, including the attenuated psychotic symptom criterion, the BLIPS criterion, and the genetic risk and functional decline criterion. COPER and COGDIS were assessed by the Schizophrenia Proneness Instrument, adult [SPI-A (40)] and children/adolescent version [SPI-CY (41)]. A detailed description of the ultra-high-risk and basic symptom criteria can be found in Table S1 in Supplementary Material. Good interrater-reliability and construct-validity (48, 49) were reported for the assessments of CHR-criteria that also possess good test–retest reliability across short periods of time and assessment modes (48–51).

#### Childhood Adversities and Trauma

The Trauma And Distress Scale [TADS (52)] is a self-report questionnaire to assess retrospectively the frequency of five types of self-reported childhood adversities and trauma: emotional neglect, physical neglect, sexual abuse, emotional abuse, and physical abuse. Each of the 43 items is rated on a 5-point Likert-scale from “never” to “almost always.” Higher values indicate more severe adversities and trauma. The TADS has been used in adolescent as well as adult samples (53, 54) and has been validated in a large general population study showing good internal consistency, inter-method reliability, and concurrent validity (52).

#### Coping

Coping-strategies were evaluated by the German Stress-Coping-Questionnaires using the version for adults [SVF-120 (42)] and children/adolescents [SVF-KJ (43)], which define coping-strategies as a person’s habitual reactions to stressful events. The frequency of each coping-strategy is rated on a 5-point Likert-scale ranging from “not at all” to “in any case.” Both versions allow the calculation of summary scores for positive and negative coping-strategies from 16 (SVF-120) and 9 (SVF-KJ) primary scales, respectively. Gender-adapted and age-adapted normative data are provided as T-values. Both age-adapted versions of the SVF have shown good internal consistency, retest reliability and construct, as well as criterion validity (42, 43, 55).

#### Competence and Control Beliefs

The German Competence and Control Beliefs Questionnaire [FKK (46)] is a 32-item questionnaire to assess a person’s generalized expectations about own competencies and courses of action (“self-concept”) as well as causal attributions of events to oneself (“internality”), to other persons (“social externality”), or to chance/situational factors (“fatalistic externality”). Each item is rated on a 6-point Likert-scale ranging from “totally false” to “totally true.” Higher values indicate a stronger tendency for the respective competence/control belief. Age-adapted normative data are provided as T-values. Studies support the internal consistency, test–retest reliability and content, construct, concurrent, as well as predictive validity of the FKK in adolescents and adults (46).

#### Depressiveness

The Beck Depression Inventory [BDI-II (56)] is a 21-item self-assessment of depressiveness in the past 2 weeks. Each item is rated on a 4-point Likert-scale as described below. The summary score excluding suicidal ideation (item 9) was used with higher scores indicating more severe depressiveness. The BDI-II has been widely used among adolescents and adults (57, 58) to assess the severity of depressive symptoms with good psychometric properties in terms of internal consistency, retest reliability as well as content, construct, concurrent, and predictive validity (56, 59–61).

#### Suicidality

Suicidality was assessed by two measures to determine suicidality-domains: suicidal ideation and suicidal risk. The “suicidal ideation” item 9 of the BDI-II (56) was used to determine suicidal ideation in the past two weeks rated on a 4-point Likert-scale ranging from “absent” (“I don’t have thoughts of killing myself”) to “severe” (“I would kill myself if I had the chance”). The “suicidality scale” of the Mini International Neuropsychiatric Interview in its version for adults [MINI (44)] and children/adolescents [MINI-KID (45)] was used to determine suicidal risk with regard to suicidal ideation, plans, and attempts. In the MINI/MINI-KID, the interviewer asks yes–no questions about the presence of suicidal ideation, plans, and attempts within the past month. Points are granted for each question answered with “yes,” while the number of points depends on the severity of the respective indicator for suicidality. The summary score was used to rate the current suicide risk as “not present” (0 points), “low” (1–8 points), “moderate” (9–16 points), or “high” (>17 points). Both instruments have shown to be reliable measures with good concurrent and predictive validity for assessing suicidality in children/adolescents and adults (44, 45, 62–65).

### Statistical Analyses

All analyses were performed using Mplus version 7.4 with the weighted least squares mean and variance adjusted estimator (WLSMV) for categorical variables (66). Data (8.5%) were missing completely at random (MCAR) as indicated by Little’s MCAR test [χ^2^(88) = 97.25, *p* = 0.235]. They were replaced through multiple imputations by creating 50 complete datasets that were used for all subsequent analyses (67).

Structural equation models were calculated to investigate the hypothesized mediation effects. Model fit was assessed by five commonly used indices: Chi-square test (χ^2^), Comparative Fit Index (CFI), Tucker–Lewis Index (TLI), root-mean-square error of approximation (RMSEA), and the Weighted Root Mean Square Residual (WRMR). To generate measurement models, latent variables were formed for adversities and trauma (emotional abuse/neglect, physical abuse/neglect, sexual abuse), coping (positive/negative coping styles), competence/control beliefs (self-concept, internality, social externality, fatalistic externality), and suicidality-domains (MINI/MINI-KID suicidality subscale; BDI-II, item 9). The summary score of the BDI-II was used as a manifest indicator for current depressiveness; presence of any CHR-criterion was treated as binary manifest variable.

Following recommendations for assessing mediation effects (68, 69), we initially tested a basic model, which postulates a significant association between the independent variable “childhood adversities and trauma” and the dependent variable “suicidality” (hypothesis 1). To examine hypotheses 2 and 3, potential mediators needed to be associated with both the independent and dependent variable as a precondition to establish a mediation effect (Figures S1–S3A,B in Supplementary Material). Significance of indirect effects was tested by calculating bootstrapped, bias-corrected confidence intervals (CIs) of the indirect effect (70). Finally, potential socio-demographic and clinical confounding variables (age, gender, educational level, current comorbid axis-I disorders) were included as covariates. Additional models were calculated to test if the relationship between adversities/trauma and suicidality was also mediated by each mediator separately (Table S2 in Supplementary Material).

## Results

### Sample Characteristics

The sample consisted of 73 CHR-patients aged between 9.5 and 35.3 years with the majority (84.9%, *n* = 62) falling within an age-range between 12 and 25 years (Table 1). 44 CHR-patients (60.3%) were younger than 18 years. Therefore, they completed the child/adolescent versions of the respective instruments, i.e., SPI-CY (41), SVF-KJ (43), and MINI-KID (45), while all other instruments were completed by the whole sample.

**Table 1:** Socio-demographic and clinical sample characteristics (*n* = 73).

Socio-demographic and clinical data		
*Age* in years, *mean (SD), median (quartiles), age categories in years, n (%)*	18.4 (4.6), 17.5 (15.7; 20.9), <12 years: 5 (6.8%); <18 years: 43 (58.9%), ≥18 years: 29 (39.7%); >25 years: 6 (8.2%)	
*Gender*, male, *n (%)*	38 (52.1%)	
*Nationality*, Swiss, *n (%)*	63 (86.3%)	
*Highest ISCED* score school (3ab), *n (%)*	15 (20.6%)	
*Functional outcome, SOFAS, mean (SD)*	61.0 (11.3)	
*Axis-I diagnoses*^a^, *n (%)*		
Current major depressive episode	12 (17.4%)	
Past major depressive episode	28 (40.6%)	
Recurrent episodes of major depression	15 (22.1%)	
Current substance use disorders	14 (20.3%)	
Current anxiety disorders	18 (24.7%)	
Past anxiety disorders	13 (19.1%)	

**Model variables**	**With missing values**	**With imputed values^b^**

*Childhood adversities and trauma*		
*TADS, mean (SD)*		
Emotional neglect	7.7 (4.9)	7.8 (4.8)
Physical neglect	5.4 (3.3)	5.4 (3.3)
Sexual abuse	1.7 (3.7)	1.7 (3.6)
Emotional abuse	6.6 (4.9)	6.7 (4.8)
Physical abuse	3.0 (3.1)	3.0 (3.1)

***Competence and control beliefs***		
*FKK, summary T-scores, mean (SD)*		
Self-concept	45.8 (9.7)	45.9 (9.7)
Internality	44.9 (10.1)	44.9 (10.0)
Social externality	49.6 (9.6)	49.5 (9.5)
Fatalistic externality	50.6 (8.7)	50.5 (8.6)

***Coping-strategies***		
*SVF, summary T-scores, mean (SD)*		
Positive coping-strategies	41.3 (11.4)	40.5 (11.8)
Negative coping-strategies	54.7 (12.9)	54.6 (12.8)

***Depressiveness***		
*BDI-II, summary score, mean (SD)*	23.3 (11.6)	23.4 (11.5)

***Suicidality***		
*BDI-II, item 9, n (%)*		
No suicidal ideation (0)	23 (31.5%)	26 (35.6%)
Mild ideation (1)	34 (46.6%)	35 (47.9%)
Severe ideation (2)	9 (12.3%)	10 (13.7%)
Very severe ideation (3)	2 (2.7%)	2 (2.7%)
*MINI/-KID, suicidality risk, n (%)*		
Absent	58 (79.5%)	62 (84.9%)
Low	5 (6.8%)	5 (6.8%)
Moderate	–	1 (1.4%)
High	5 (6.8%)	5 (6.8%)

*APS, attenuated psychotic symptom criterion; BDI-II, Beck Depression Inventory (56); BLIPS, brief limited intermittent psychotic symptom criterion; COGDIS, cognitive disturbances criterion; COPER, cognitive-perceptive basic symptoms criterion; FKK, Competence and Control Belief Questionnaire (46); GRFD, genetic risk and functional decline criterion; ISCED, International Standard Classification of Education; MINI/-KID, Mini-Neuropsychiatric Interview for adults and children (44, 45); SOFAS, Social and Occupational Functioning Assessment Scale (71); SVF, Stress-Coping-Questionnaires for adults and children (42, 43); TADS, Trauma And Distress Scale (52)*.

*^a^Multiple diagnoses were possible; ≥5% of individuals fulfilled the diagnostic criteria for the respective mental disorder*.

*^b^For categorical variables, the most frequent imputation pattern is reported. No data were missing for clinical high-risk criteria*.

Table 1 shows sample characteristics and summary statistics for each model variable. With regard to childhood adversities and trauma, 35.8% (*n* = 24) CHR-patients reported clinically relevant levels of emotional abuse and emotional neglect, i.e., they scored more than 1 SD above the respective mean of the normative data provided (52); 31.3% (*n* = 21) reported physical neglect, 23.9% (*n* = 16) physical abuse and 21.5% (*n* = 14) sexual abuse. Furthermore, CHR-patients frequently demonstrated a lack of positive coping-strategies (48.4%, *n* = 30) and an excessive use of negative strategies (30.8%, *n* = 20) according to the test norms (42, 43). Moreover, they also showed deficits in competence and control beliefs in terms of a negative self-concept (28.8%, *n* = 19), low levels of internal attributions (33.3%, *n* = 22) as well as an excessive use of social external attributions (10.6%; *n* = 7) and fatalistic external attributions (15.2%, *n* = 10) according to the test norms (46). In addition, CHR-patients reported on average moderate levels of depressiveness with 66.2% (*n* = 45) having at least mild levels of suicidal ideation in the past 2 weeks as assessed by the BDI-II-item (56). Ten CHR-patients (13.6%) had at least a low risk for suicidality in the past month as assessed by the MINI/MINI-KID (44, 45). All of them also reported at least a minimal level of suicidal ideation.

### Childhood Adversities/Trauma and Suicidality (Model 1)

**Figure 1:**
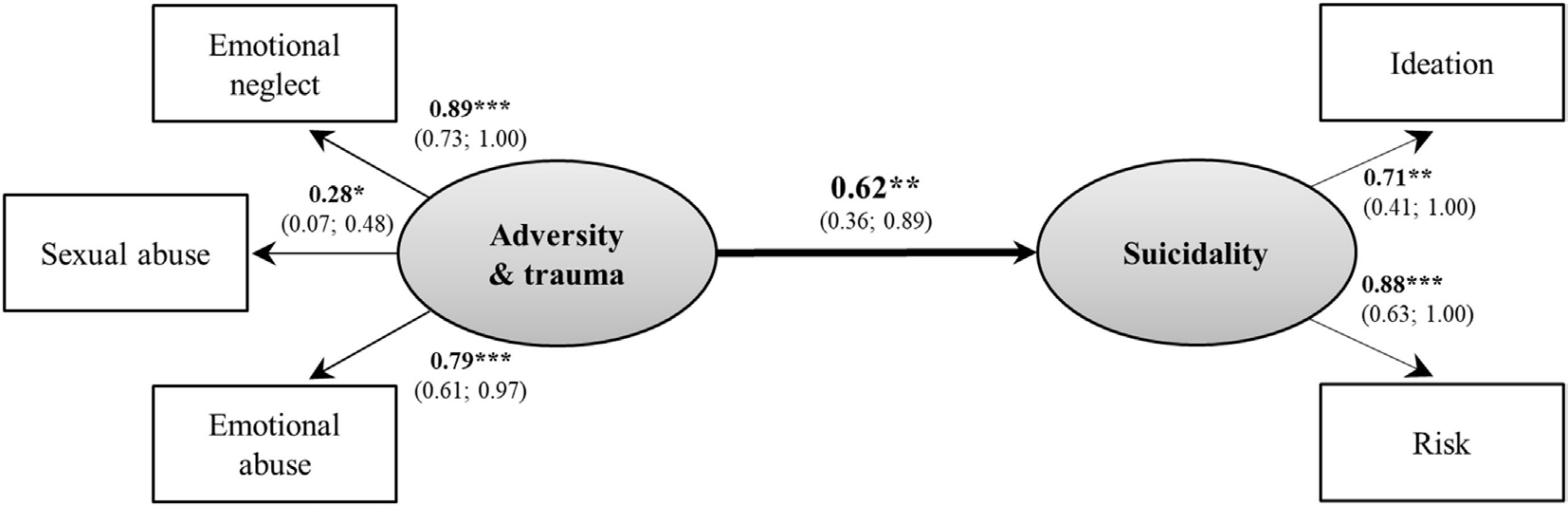
Basic model between childhood adversities/trauma and suicidality. Model fit indices: χ^2^(4) = 3.61, *p* = 0.461; Comparative Fit Index = 1.00; Tucker–Lewis Index = 1.02; root-man-square error of approximation = 0.00, *p* = 0.552; Weighted Root Mean Square Residual = 0.34. Rectangles present observed manifest variables, ovals unobserved latent variables; values are standardized path coefficients and 95% confidence intervals of parameter estimates. *p < 0.05, **p < 0.01, ***p < 0.001.

Bivariate correlations among the measures are shown in Table 2. As expected, we found significant associations of several domains of childhood adversities and trauma, namely emotional abuse as well as neglect and sexual abuse, with both suicidality-domains. Consistent with our first hypothesis, childhood adversities and trauma were significantly associated with suicidality (β = 0.50, *p* = 0.003) with adequate model fit [χ^2^(13) = 17.15, *p* = 0.192; CFI = 0.95; TLI = 0.91; RMSEA = 0.07, *p* = 0.349; WRMR = 0.51]. Dropping the two domains of childhood adversities and trauma that were uncorrelated with either suicidality-domain (Table 2), i.e., physical abuse and neglect, from the model resulted in an excellent model fit (Figure 1), and the association between childhood adversities and trauma and suicidality was significant again (β = 0.50, *p* = 0.002). Consequently, we reduced the latent variable childhood adversities and trauma to three indicators (emotional abuse and neglect, sexual abuse) in subsequent models.

**Table 2:** Bivariate correlations between model variables with imputed values.

Model variables	1	2	3	4	5	6	7	8	9	10	11	12	13	14	15	16	17
1. Emotional abuse	–																
2. Emotional neglect	0.79**	–															
3. Physical abuse	0.45**	0.57**	–														
4. Physical neglect	0.59**	0.69**	0.64***	–													
5. Sexual abuse	0.15	0.20	0.38**	0.11	–												
6. Positive coping	−0.25*	−0.35**	−0.06	−0.16	−0.14	–											
7. Negative coping	0.24	0.24	0.20	0.24	0.32*	0.15	–										
8. Self-concept	−0.31*	−0.25*	0.11	0.05	−0.19	0.52**	−0.24	–									
9. Internal beliefs	−0.14	−0.18	0.03	0.05	−0.05	0.46**	0.12	0.34**	–								
10. Social-external beliefs	0.33**	0.20	0.07	0.13	0.22	−0.17	0.27*	0.39**	−0.01	–							
11. Social-fatalistic beliefs	0.32**	0.24*	0.24	0.17	0.21	−0.38**	0.20	−0.50**	0.03	0.44**	–						
12. Depressiveness	0.47**	0.53**	0.25*	0.23	0.36**	−0.57**	0.17	−0.58**	−0.24*	0.30*	0.44**	–					
13. Cognitive disturbances	0.31*	0.27	0.09	0.09	−0.19	−0.27	0.03	−0.11	−0.17	0.10	0.33*	0.20	–				
14. Cognitive-perceptive basic symptoms	0.39*	0.42**	0.34*	0.43**	−0.23	−0.10	0.15	−0.15	0.05	0.22	0.38*	0.12	0.46**	–			
15. Attenuated psychotic symptoms	0.03	0.00	0.26*	−0.19	0.02	−0.12	−0.06	−0.17	−0.21	−0.16	−0.06	0.13	−0.14	−0.44**	–		
16. Suicidal ideation^a^	0.33**	0.32**	0.05	0.06	0.21	−0.27*	0.10	−0.25*	−0.18	0.20	0.21	0.68**	0.32*	0.02	0.04	–	
17. Suicide risk^b^	0.23	0.29*	0.19	0.11	0.31*	−0.28*	0.08	−0.27*	−0.25*	0.18	0.15	0.51**	0.35*	0.14	−0.03	0.45**	–

*^a^Assessed by the Beck Depression Inventory II, item 9 (BDI-II; 56)*.

*^b^Assessed by the Mini-Neuropsychiatric Interview for adults and children (MINI/MINI-KID; 44, 45)*.

*p < 0.05.

**p < 0.01.

***p < 0.001.

### Psychological Mediators between Childhood Adversities/Trauma and Suicidality (Model 2)

**Figure 2:**
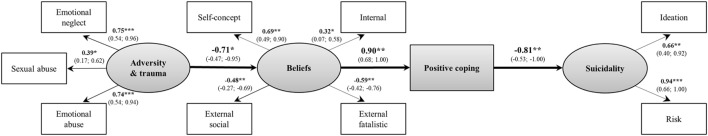
Psychological mediators between childhood adversities/trauma and suicidality. Model fit indices: χ^2^(33) = 43.41, *p* = 0.106; Comparative Fit Index = 0.90; Tucker–Lewis Index = 0.87; root-mean-square error of approximation = 0.06, *p* = 0.300; Weighted Root Mean Square Residual = 0.61. Standardized indirect effect, IE = 0.54; 95% confidence intervals (Cis) = 0.27; 0.82; *p* = 0.005. Rectangles present observed manifest variables, ovals unobserved latent variables; values are standardized path coefficients and 95% CIs of parameter estimates. *p < 0.05, **p < 0.01, ***p < 0.001.

A lack of positive coping-strategies was significantly associated with both emotional abuse and neglect, and suicidality-domains, while negative coping-strategies were unrelated to either suicidality-domain and to positive coping-strategies (Table 2). Consequently, no latent coping-variable could be formed, and negative coping-strategies were dropped from the model. With regard to competence/control beliefs, a negative self-concept was significantly associated with emotional abuse as well as neglect and suicidality-domains. An excessive use of social and fatalistic external beliefs was significantly correlated with both emotional abuse and neglect; a lack of internal beliefs with suicide risk. In line with our second hypothesis, both positive coping-strategies and dysfunctional competence/control beliefs functioned as mediators between childhood adversities as well as trauma and suicidality as indicated by significant indirect effects and adequate model fit (Figure 2, Figure S4).

### Final Model with Symptomatic and Psychological Mediators (Model 3)

Testing our third hypothesis, including symptom levels as additional mediators of the identified indirect effects, higher levels of depressiveness were significantly correlated with various domains of childhood adversities and trauma, both suicidality-domains, and psychological mediators (Table 2). While presence of COGDIS was related to emotional abuse, COPER was related to emotional/physical abuse and emotional/physical neglect. However, only COGDIS was significantly correlated with both suicidality-domains and, therefore, included in the model.

Presence of any attenuated psychotic symptom was significantly associated with physical abuse but unrelated to either suicidality-domain (Table 2). With regard to single attenuated psychotic symptoms, we found a significant relationship between the presence of any persecutory idea (P2-item of the SIPS) and emotional abuse (*r* = 0.25, *p* = 0.042) as well as between perceptual abnormalities as well as attenuated hallucinations (P4-item of the SIPS) and suicidal ideation (*r* = 0.29, *p* = 0.017). However, SIPS-P4 was unrelated to all other model variables, thus making a mediation effect impossible. When used as a predictor instead of a mediator variable, SIPS-P4 was not significantly associated with suicidality in the model (*r* = 0.29, *p* = 0.071), and, therefore, dropped from the final model. Associations between presence of BLIPS and any other model variable could not be investigated because only three CHR-patients (4.1%) met this criterion.

**Figure 3:**
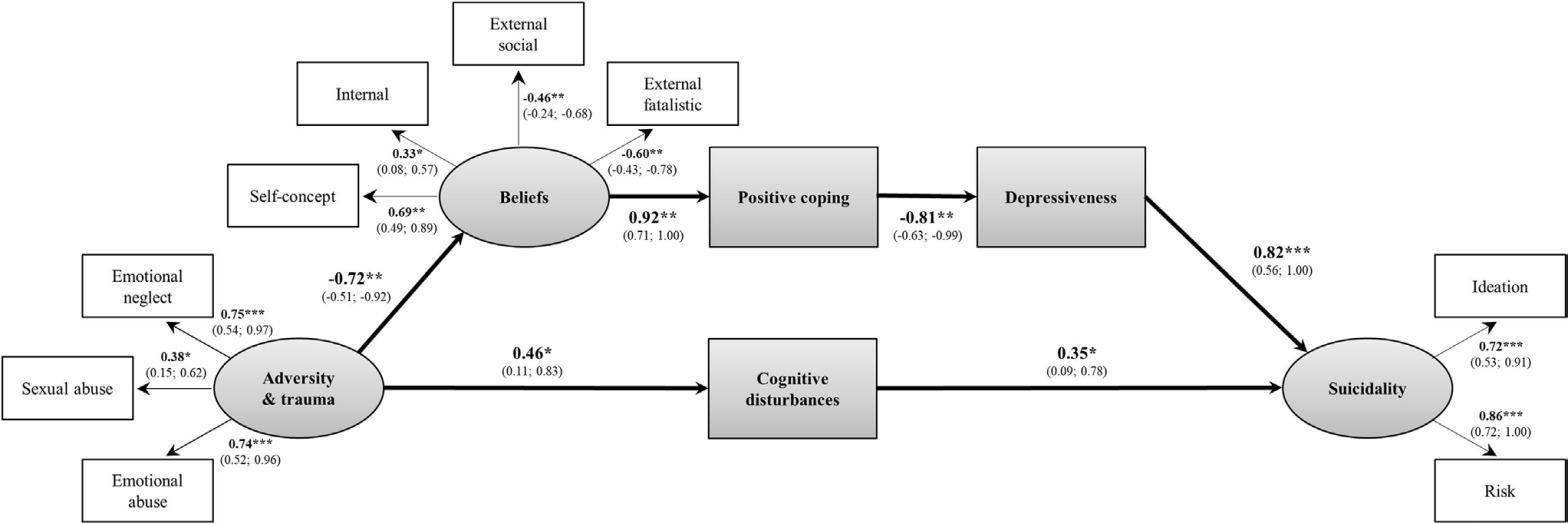
Psychological and symptomatic mediators between childhood adversities/trauma and suicidality. Model fit indices: χ^2^(51) = 59.68, *p* = 0.190; Comparative Fit Index = 0.95; Tucker–Lewis Index = 0.94; root-mean-square error of approximation = 0.046, *p* = 0.496; Weighted Root Mean Square Residual (WRMR) = 0.59. Standardized indirect effect through beliefs–coping–depressiveness: IE = 0.44; 95% CIs = 0.10; 0.78; *p* < 0.001 and through cognitive disturbances: IE = 0.16, 95% confidence intervals (Cis) = 0.01; 0.34; *p* = 0.045. Rectangles present observed manifest variables, ovals unobserved latent variables; values are standardized path coefficients values are standardized path and 95% CIs of parameter estimates. *p < 0.05, **p < 0.01, ***p < 0.001.

Thus, two independent mediation pathways were detected in the final model. One pathway ran from experiences of childhood adversities and trauma through more dysfunctional competence/control beliefs and a lack of positive coping-strategies to more depressiveness and from there to an increased risk for suicidality (Figure 3). This indirect effect was significant. The second pathway was weaker and led from experiences of childhood adversities and trauma through an increased probability to meet COGDIS to an increased risk for suicidality. This indirect effect was also significant. The same pattern of results was observed when the sequence of the psychological mediators was changed (Figure S5). The final model had a good fit to the data. Additional structural equation models, which included each mediator (i.e., dysfunctional beliefs, lack of positive coping, depressiveness, and COGDIS) separately, could confirm the relevance of each variable to explain this relationship between adversities/trauma and suicidality as indicated by significant indirect effects (Table S2 in Supplementary Material).

No changes were suggested by the modification indices that would turn the single pathway from childhood adversities and trauma on suicidality through psychological mediators into a dual pathway (e.g., through a separate path for coping and competence/control beliefs). None of the included covariates had a significant effect on suicidality and all of the identified pathways remained stable when they were included in the final model.

## Discussion

The results of this study extend the current literature on psychological and symptomatic mediation effects between childhood adversities and trauma and suicidality to patients at CHR for psychosis as well as to the effects of attenuated psychotic symptoms and basic symptoms. We found in our first model that childhood adversities and trauma were significantly related to suicidality. This relationship was mainly conveyed by sexual and emotional abuse as well as emotional neglect, while inclusion of physical abuse and neglect led to worse model fit. Only one study so far (72) has investigated this relationship in CHR-patients; it revealed no effect of trauma on the number of suicide attempts. Yet, this study had assessed only trauma but not adversities and only suicide attempts but not suicidal ideation, which we found to be highly prevalent and interrelated in our study. Furthermore, trauma was treated as a single entity (72), which may have masked actual associations because our results suggest a differential relationship of different domains of childhood adversities and trauma with suicidality. With regard to our data, the lack of an association between physical adversities as well as trauma and suicidality was not explained by a lower prevalence of physical compared to emotional adversities/trauma because the most infrequent domain sexual abuse was nevertheless significantly associated with suicidality. This is well in line with the literature (8, 73). Furthermore, the strong effect of emotional adversity and trauma in our study corresponds to previous studies that investigated the differential effects of various domains of childhood adversities and trauma and reported emotional neglect to have the strongest association with suicidality (74, 75). This is consistent with the assumption of the interpersonal–psychological theory of suicide (24) that adversities and trauma increase the desire for suicide by augmenting feelings of perceived burdensomeness and decreased belongingness. Such feelings are assumed to result from all forms of adversities and trauma but seem to be especially amplified in the presence of high levels of negative emotions, which are an integral part of emotional neglect and abuse (23).

With regard to the second model, as expected the relationship between childhood adversities as well as trauma and suicidality was mediated by dysfunctional competence/control beliefs and coping. Yet, unexpectedly, the role of coping on suicidality was only conveyed by positive coping-strategies but not additionally by negative coping-strategies, which were not significantly correlated with each other. This is in contrast to previous results (76, 77) and might be caused by the fact that our CHR-patients rather reported a lack of positive coping-strategies than an excessive use of negative strategies. Furthermore, severe forms of trauma, such as sexual abuse that were predominately linked to an excessive use of negative coping-strategies were rare in our sample. In addition, our assessment was not limited to abuse but included neglect, which is thought to exert a less detrimental effect on coping and suicidality than abuse (78).

With regard to the sequence of competence/control beliefs and coping, both alternative models (competence/control beliefs first and coping second as well as *vice versa*) were supported by our data. Models placing competence/control beliefs first posit that individuals, who perceive themselves to be less competent and events to be predominantly controlled by external factors, are less likely to initiate and sustain positive coping behavior (34, 79). Models with the reverse sequence, however, state that exposure to childhood adversities and trauma has an adverse effect on cognitive development (80), which results in poor coping skills (81). Ineffective coping skills lead to long-standing difficulties when faced with future stressors, which increases the risk to develop dysfunctional competence/control beliefs as well as depressiveness and suicidality (77, 82–84). Our results argue for a more complex and synergistic view in that both constructs mutually influence each other, i.e., beliefs about low controllability and few competences reduce the probability to apply positive coping-strategies, which leads to even more dysfunctional beliefs and *vice versa*. The high path coefficients of the relationship between competence/control beliefs and coping in our models suggest that both constructs represent a higher-order phenomenon known as “coping-efficacy” (85), which refers to an individual’s beliefs about the efficacy of coping in the future based on previous coping-experiences. In line with our results, coping-efficacy mediated the relationship between childhood adversities as well as trauma and depression (86) and was linked to suicidality (87).

With regard to the other mediators and covariates in model 3, none of the assumed covariates had a significant direct relationship to suicidality, while of the symptomatic mediators only depressiveness and COGDIS but not attenuated psychotic symptoms and COPER became significant. Thereby, depressiveness followed competence/control beliefs and coping and had a direct impact on suicidality. COGDIS was an independent mediator between childhood adversities as well as trauma and suicidality, introducing a second, yet weaker pathway.

The first pathway implies that childhood adversities and trauma act as a catalyst in the suicidal process by triggering the development of dysfunctional competence/control beliefs. This belief pattern is often referred to as “hopelessness” (46) and may set back the application of positive coping-strategies, which then leads to depressiveness and finally to suicidality. This model is consistent with integrated hopelessness and interpersonal-psychological theories of suicidality and related empirical findings. They suggest that childhood adversities and trauma confer risk for the development of a negative and hopeless cognitive style (defined as low control beliefs for negative events and negative self-evaluations) with the consequence that a person is hopeless about the future and develops depressiveness and suicidality (7, 88). In line with our results, in particular emotional abuse and neglect were reported to be associated with the development of such a negative cognitive style, possibly because the abuser directly supplies the abused individual with self-blaming statements (e.g., “I believe that I am a bad person”; TADS-item 14) often involved in suicidal ideation (7). Furthermore, the relationship between emotional adversities/trauma and poor coping reported in our and other studies may be explained by the fact that maltreating parents/care-givers are likely inappropriate role models due to their own coping deficits, who cannot provide a supportive environment to learn adequate coping-strategies (7, 76). This pathway has often been described in the development of depression (89, 90) and may reflect the high percentage of depressiveness in our sample and in CHR-patients in general (91).

With regard to attenuated psychotic symptoms, presence of any attenuated psychotic symptom was significantly associated with childhood adversities and trauma in terms of physical abuse but not with suicidality. With regard to single attenuated psychotic symptoms, persecutory ideas were significantly associated with emotional abuse. This is in line with results in patients with psychosis (92) that also found this specific relationship. However, only perceptual abnormalities and attenuated hallucinations were significantly associated with suicidality. This specific association is in accordance with studies on psychotic-like experiences (29, 77) and on patients with psychosis (93). However, no significant mediation effect of any attenuated psychotic symptom between childhood adversities as well as trauma and suicidality was found in this study. This suggests that childhood adversities and trauma may not be specific for the development of psychotic symptoms as other symptomatic mediators, in particular depressiveness, were more influential in conveying the link between adversities/trauma and suicidality. This is supported by the result that childhood adversities and trauma contribute to a shared vulnerability for both the development of psychosis and in particular of depression, with the latter being the strongest predictor of suicidality (94). Furthermore, other factors may have a stronger impact on suicidality than (attenuated) psychotic symptoms, which is supported by the result that suicidality in patients with first-episode psychosis is highest in the remission phase (95).

In light of the missing mediation effect through attenuated psychotic symptoms in our study and inconclusive results about the relationship between higher cognitive functions and suicidality in schizophrenia (10), the role of COGDIS is especially noteworthy. COGDIS includes subtle, subclinical disturbances in thinking that are self-experienced with immediate full insight into their abnormal nature (12). One possible explanation focuses on the full insight when experiencing basic symptoms. This was reported to lead to increased levels of affective tension that decrease when (attenuated) psychotic symptoms develop (96). Therefore, basic symptoms may be more closely linked to suicidality than attenuated psychotic symptoms. Furthermore, it has been suggested that insight may result in a negative change in self-image and/or an exaggerated awareness of consequences related to a possible mental disorder and feelings of stigma. Thereby high levels of insight, in particular prior to treatment, increase risk for suicidality (10, 97). This might link our results on COGDIS to findings of increased levels of social isolation and feelings of burdensomeness and belongingness as the main predictors of suicidality (24, 30). While there is a large overlap between COGDIS and COPER, the latter was only significantly associated with childhood adversities and trauma but not with suicidality. This may be due to the lower specificity of COPER in predicting conversion to psychosis (98, 99).

Apart from the strengths of our study (e.g., detailed examination of adversity-/trauma- and CHR-domains), some limitations have to be discussed. First, we used only a cross-sectional design, which does not allow firm conclusion about the causality between the investigated variables. In particular, it was not possible to disentangle cause and effect or continuous interaction between competence/control beliefs and coping, which highlights the need for more longitudinal studies. Furthermore, due to the large age-range, younger individuals might still be exposed to childhood adversities and trauma in contrast to older ones, which also challenges the assumption of a unidirectional sequence of the model variables. In addition, although age was not significantly associated with suicidality as the outcome of interest in our model and did not affect the relationships between the model variables when included as a covariate, future studies with a more homogeneous sample or with larger samples-sizes in each age-group should be conducted to test for potential age-effects using more sophisticated analyses (e.g., multiple-group comparisons). Yet, our age-range might also be an advantage as the effect of recent or even still on-going childhood adversities and trauma might be even stronger than the effect of past ones. Furthermore, we used a theory-driven approach, which clearly suggests that childhood adversities and trauma contributes to the formation of dysfunctional belief- and coping-pattern and the development of depressiveness and suicidality. Second, we were interested in answering the question how childhood adversities and trauma and suicidality are linked to each other. However, alternative models may fit the data equally well, e.g., that high levels of self-efficacy and positive coping moderate the relationship between childhood adversities and trauma and suicidality (100). Together with the fact that we made *post hoc* modifications on the model, this clearly warrants a cross-validation and comparison of alternative models in a larger sample. This seems also indicated as our sample size was rather small within the context of structural equation modeling, which is associated with a higher risk of detecting spurious effects as well as of not detecting true but small effects and with larger CIs for estimation of the indirect effect limiting the generalizability of our results (69). Thus, our results and conclusions are only preliminary and need to be interpreted cautiously. Third, the fact that we assessed depressiveness and suicidal ideation with the same measurement scale may have increased the correlation between them. However, we did not rely on a single item to assess suicidality as many previous studies but additionally applied the suicidality subscale of a structured clinical interview. Both instruments have demonstrated good predictive validity to assess suicidality in adolescents and adults (62–65).

Despite these limitations, our study has important clinical implications. It suggests that interventions to prevent suicidality in CHR-patients should focus on both reducing COGDIS and disrupting the detrimental cascading effect through poor coping skills, dysfunctional belief pattern, and depressiveness as early as possible. In order to identify CHR-patients at risk for suicidality, childhood adversities and trauma as well as competence/control beliefs and coping styles should be routinely assessed, monitored, and targeted if necessary. Trauma-focused interventions for CHR-patients should be supplemented by interventions to enhance positive beliefs about own competencies and controllability over events as well as to increase the repertoire of positive coping-strategies, such as re-attribution and coping-strategy enhancement techniques (101, 102) to increase resilience against depressiveness and suicidality (88, 100).

In summary, our results identified psychological and symptomatic mechanisms that contribute to the explanation why individuals exposed to childhood adversities and trauma develop suicidality. Therefore, these mediators should be monitored regularly and should be targeted therapeutically in addition to trauma-focused interventions as early as possible to enhance resilience against suicidality.

## Ethics Statement

The authors assert that all procedures contributing to this work comply with the ethical standards of the relevant national and institutional committees on human experimentation and with the Helsinki Declaration of 1975, as revised in 2008, as well as with the Swiss Federal Act on Research involving Human Beings as of 2011. All participants and, in case of minors, their care-givers were evaluated for their capacity to provide informed consent before giving written informed consent to the further use of their clinical data for research. The ethics committee of the University of Bern approved the procedure.

## Author Contributions

SS, FS-L, BS, and DH designed the study. CM and NI managed the data and the recruitment. All authors managed the literature searches and analyses. SS, NI, and BN undertook the statistical analyses. SS and FS-L wrote the first draft of the manuscript. All authors contributed to and have approved the final manuscript.

## Conflict of Interest Statement

The authors declare that the research was conducted in the absence of any commercial or financial relationships that could be construed as a potential conflict of interest.
