# Supplementary material for: Mediators Linking Childhood Adversities and Trauma to Suicidality in Individuals at Risk for Psychosis
Source: Front Psychiatry. 2017 Nov 20;8:242. doi: 10.3389/fpsyt.2017.00242 (PMC5715383; doi:10.3389/fpsyt.2017.00242)
Supplement: Supplementary file 1 [file table_1.docx]

Supporting information to:

**Mediators linking childhood adversities and trauma to suicidality in individuals at risk for psychosis**

Schmidt SJ, Schultze-Lutter F, Bendall S, Groth N, Michel C, Inderbitzin N, Schimmelmann BG, Hubl D, Nelson B

**Table S1.** Ultra-high risk (UHR) and basic symptom (BS) criteria to detect a clinical high risk state of psychoses

| ***UHR criterion ‘Attenuated Psychotic Symptoms’ (APS)***  ⮊ At least any 1 of the following 5 symptoms with a SIPS^1^ score of ‘3’ to ‘5’:   - ***unusual thought content /*** non-paranoid, non-grandiose ***delusional ideas (P1)*** not held with full conviction, including magical ideation, non-paranoid ideas of reference not immediately rectified by cognition, and attenuated “Ich-Störungen” - ***suspiciousness / persecutory ideas (P2)*** not held with full conviction - ***grandiose ideas (P3)*** of special powers or missions not held with full conviction - ***perceptual abnormalities / hallucinations (P4)*** with remaining insight in their abnormal nature, incl. schizotypal phenomena such as sensing the presence of something/someone, perceiving moving shadows in the periphery of the visual field or unusual bodily perceptions - ***disorganized communication (P5)*** and speech that is still comprehensible and responds to structuring in the interview   ⮊ First occurrence or worsening (in terms of an increase in conviction / loss of insight and/or of an increased impact on behaviour) within the past 12 months.  ⮊ At least weekly occurrence within past month. |
| --- |
| ***UHR criterion ‘Brief Limited Intermittent Psychotic Symptoms’ (BLIPS)***  ⮊ At least any 1 of the above 5 symptoms (P1-P5) with a SIPS score of ‘6’, i.e. temporarily held with full conviction or with complete lack of insight  ⮊ Psychotic level of intensity, i.e., a score of ‘6’ was reached within past 3 months.  ⮊ At least present for several minutes per day at a frequency of at least once per month. |
| ***BS criterion ‘Cognitive-Perceptive Basic Symptoms’ (COPER)***  ⮊ At least any 1 of the following 10 BS:   - ***thought interference (D9)***: Irrelevant, emotionally neutral thoughts with no special meaning and no association with the intended thought are intruding on and disturbing the young person’s train of thought, without it being lost. - ***thought perseveration (C2)***: A kind of thought interference in that intruding emotionally neutral and irrelevant thoughts or images occur not just once but repeatedly. - ***thought pressure (D10)***: A self-reported ‘chaos’ of thoughts in that successively occurring thoughts are not linked by any common thread, and are completely unrelated to each other or to the young person’s intended line of thought. - ***thought blockages (D15)***: Sudden interruption in the flow of thoughts, or experiences of the mind suddenly going blank, of a fading (slipping) of thoughts or of losing the thread of thoughts, with the original topic being recalled subsequently or lost completely. - ***disturbance of receptive speech (D11)***: A disturbance in the understanding of simple everyday words. When reading or listening to others, the young person struggles to comprehend the meaning of words, word sequences or sentences, even if the young person concentrates on the text or speech and has perceived it accurately. - ***decreased ability to discriminate between ideas and perception, fantasy and true memories (B2)***: A self-recognized difficulty in locating the source of an experience/memory (external vs. internal mental) that results in an inability to immediately distinguish between imaginations and perception, or pure fantasy and true memories. - ***unstable ideas of reference (B2)***: Subjective, subclinical experiences of self-reference for that no explanation outside own mental processes are sought, and that is immediately overcome. - ***derealisation (O8)***: A change in how one relates emotionally to the environment, which is experienced commonly as an estrangement and detachment from the visual world, or rarely as an increased emotional affinity for the environment. - ***visual perception disturbances (O4)*** (excl. blurred vision and hypersensitivity to light): Misperceptions of aspects of the visual field while the young person is fully aware of their true appearance and, therefore, attributes his or her misperception to a problem with eye sight or mental processes. - ***acoustic perception disturbances (O5)*** (excl. hypersensitivity to sounds/noises): Misperceptions of acoustic stimuli while the young person is fully aware of the true sound and, therefore, tends to attribute his or her misperception to a problem with hearing or mental processes.   ⮊ First occurrence or significant increase in frequency ≥12 months ago  ⮊ Occurrence of at least ‘several times in a month or weekly’ within the past 3 months, i.e. a SPI-A score of at least ‘3’. |
|  |
| ***BS criterion ‘Cognitive Disturbances’ (COGDIS)***  ⮊ At least any 2 of the following 9 BS:   - ***inability to divide attention (B1)***: A difficulty in dealing with demands that involve more than one sensory modality at a time and thus does not concern demands that would require quick switching of attention. - ***captivation of attention by details of the visual field (O7)***: Domination of the visual field by a random single aspect of it that captures the young person’s whole attention, impedes paying attention to other aspects and causes difficulties in turning away from it. - ***thought interference*** (see COPER) - ***thought pressure*** (see COPER) - ***thought blockages*** (see COPER) - ***disturbance of receptive speech*** (see COPER) - ***disturbance of expressive speech (C5)***: A subjective difficulty in verbal fluency and clarity of expression, with words required to express simple ideas being not forthcoming or delayed. - ***disturbances of abstract thinking (O3)***: Deficits in the comprehension of any kind of abstract, figurative or symbolic phrases or content, as well as the phenomena of ‘concretism’ (a limitation of the ability to go beyond the literal meaning of words, sentences or phrases).captivation of attention by details of the visual field (O2) - ***unstable ideas of reference*** (see COPER)   ⮊ Occurrence of at least ‘several times in a month or weekly’ within the past 3 months, i.e. a SPI-A score of at least ‘3’. |
| *Note*: A general requirement of BS is their novelty, i.e., their report as a disruption in a person’s “normal” self. Self-recognized aberrations in mental processes that have always been present in the same frequency, i.e., in a trait-like manner, can be rated in SPI-A^2,3^ (rating of “7”) but are not accounted for as BS in the strict sense and, consequently, do not contribute to BS criteria. More in-depth definitions of BS as well as example statements of patients and example questions for their assessment are provided in the SPI-A, orderable at [www.fioriti.it](http://www.fioriti.it). |

UHR criteria were originally developed with the explicit aim of detecting an imminent risk for psychoses, i.e., persons at risk for developing a first-episode within the next 12 months.^4^ In contrast, BS criteria (Table S1) were developed to detect the emerging psychotic disorder as early as possible, desirably before the onset of functional decline.^4,5^

Basic symptoms (BS) were conceptualised as the earliest primarily self-experienced psychopathological correlates of the physiological disturbances of information processing underlying the development of psychosis that develops on the basis of and partly in reaction to them.^6^

| Mediators between  adversity/trauma and suicidality | Standardized path-coefficients and  indirect effects with  95% confidence intervals | Model fit |
| --- | --- | --- |
| Positive coping | Adversity/trauma 🡪 positive coping:  β=-0.64** (-0.84; -1.00)  Positive coping 🡪 suicidality:  β=-0.84* (-0.53; -1.00)  **Indirect effect: 0.54* (0.28; 0.80)** | χ^2^_(8)_=12.25, *p*=0.140  RMSEA=0.08, *p*=0.240  CFI=0.94  TLI=0.88  WRMR=0.53 |
| Beliefs  (self-concept, internal, external social, external fatalistic) | Adversity/trauma 🡪 beliefs:  β=--0.75** (-0.51; -0.98)  Dysfunctional beliefs 🡪 suicidality:  β=-0.78* (-0.52; -1.00)  **Indirect effect: 0.58* (0.33; 0.84)** | χ^2^_(25)_= 21.14, *p*=0.349  RMSEA=0.03, *p*=0.583  CFI=0.98  TLI=0.97  WRMR=0.52 |
| Depressiveness | Adversity/trauma 🡪 depressiveness:  β=0.65*** (0.46; 0.84)  Depressiveness 🡪 suicidality:  β=0.97*** (0.82; 1.00)  **Indirect effect: 0.64** (0.41; 0.86)** | χ^2^_(8)_=7.33, *p*=0.501  RMSEA=0.00, *p*=0.628  CFI=1.00  TLI=1.01  WRMR=0.35 |
| Cognitive disturbances | Adversity/trauma 🡪 cognitive disturbances: β=0.57* (0.32; 0.83)  Cognitive disturbances 🡪 suicidality:  β=0.78** (0.45; 1.00)  **Indirect effect: 0.45* (0.13; 0.71)** | χ^2^_(8)_=12.10, *p*=0.147  RMSEA=0.08, *p*=0.249  CFI=0.94  TLI=0.89  WRMR=0.60 |

**Table S2.** Results of structural equation models with a single mediator variable Note.

*Note:* **p<0.05,* ***p*<.01, ****p*<.001

**References Supplementary Material**

1. McGlashan T, Walsh B, Woods S. *The Psychosis-Risk Syndrome. Handbook for Diagnosis and Follow-up*. New York: Oxford University Press (2010). 256 p.
2. Schultze-Lutter F, Addington J, Ruhrmann S, Klosterkötter J. *Schizophrenia Proneness Instrument, Adult Version (SPI-A).* Rome: Fioriti (2007).
3. Schultze-Lutter F. Subjective symptoms of schizophrenia in research and the clinic: The basic symptom concept. *Schizophr Bull* (2009) **35**(1):5-8. doi:10.1093/schbul/sbn139
4. Phillips LJ, Yung AR, McGorry PD. Identification of young people at risk of psychosis: validation of Personal Assessment and Crisis Evaluation Clinic intake criteria. *Aust N Z J Psychiatry* (2000) **34**:S164-9. doi:10.1046/j.1440-1614.2000.00798.x
5. Schultze-Lutter F. “First signs of emerging psychosis”. In: A Riecher-Rössler A, PD McGorry PD, editors. *Early Detection and Intervention in Psychosis - State of the Art and Future Perspectives; Key Issues in Mental Health*. Karger (2016). p. 29-41.
6. Schultze-Lutter F, Debbané M, Theodoridou A, Wood SJ, Raballo A, Michel C et al. Revisiting the basic symptoms concept: toward translating risk symptoms for psychosis into neurobiological targets. *Front Psychiatry* (2016) **7**:9. doi:10.3389/fpsyt.2016.00009
