# Supplementary material for: Mediators Linking Childhood Adversities and Trauma to Suicidality in Individuals at Risk for Psychosis
Source: Front Psychiatry. 2017 Nov 20;8:242. doi: 10.3389/fpsyt.2017.00242 (PMC5715383; doi:10.3389/fpsyt.2017.00242)
Supplement: Supplementary file 2 [file presentation_1.pdf]

Supporting information to:

Psychological and symptomatic mediators linking childhood adversities and trauma to suicidal behavior in adolescents and adults at clinical high risk for psychosis

Including all hypothesized models and all alternative models tested

Schmidt SJ, Schultze-Lutter F, Bendall S, Groth N, Michel C, Inderbitzin N, Schimmelmann BG, Hubl D, Nelson B

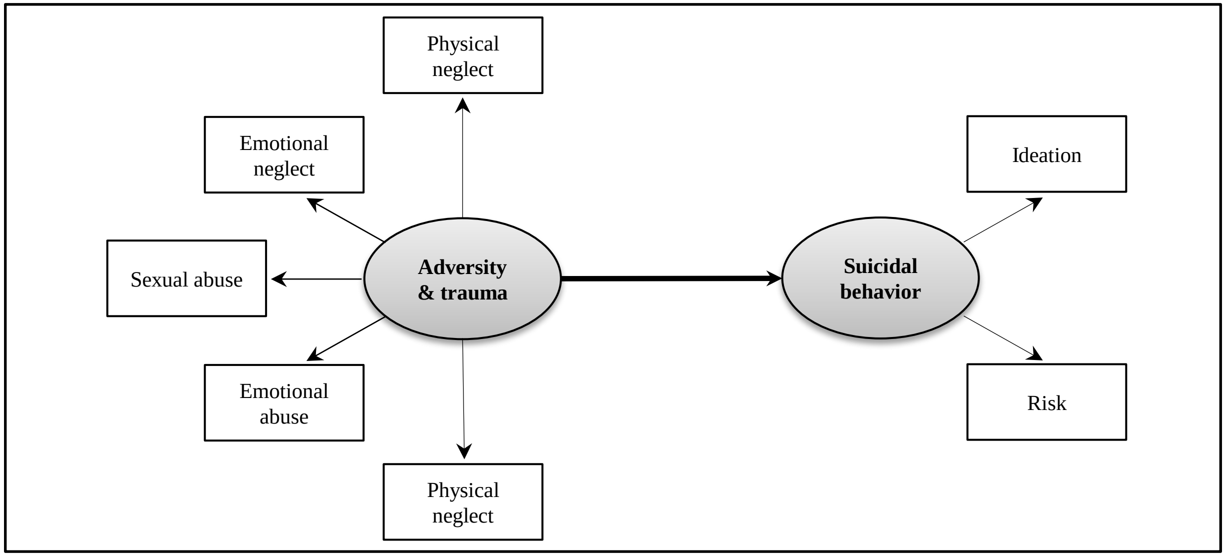

Figure S1. Basic model between childhood adversities/trauma and suicidal behavior.

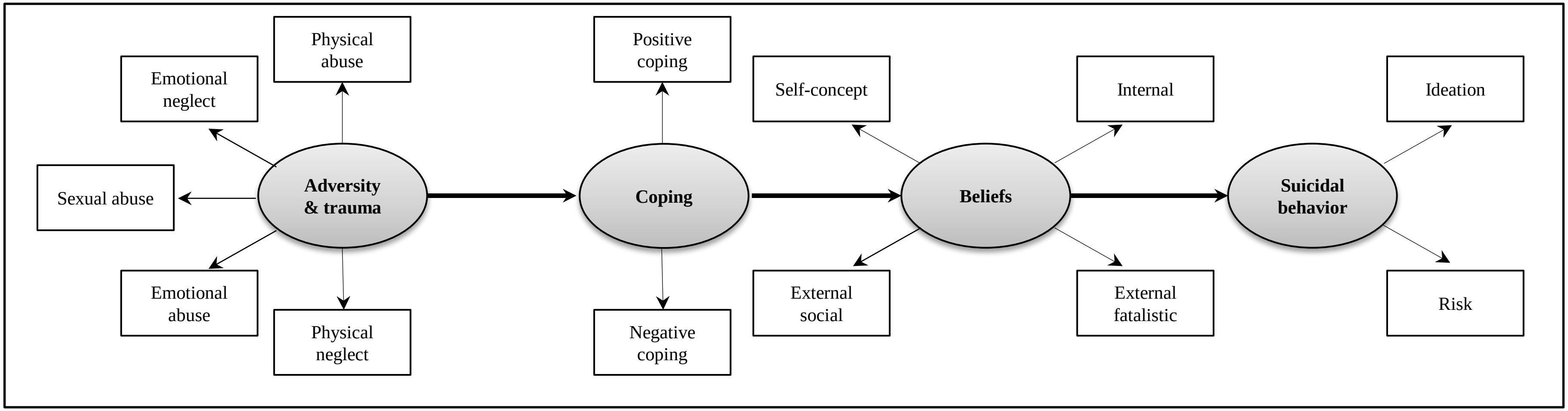

Figure S2a. Psychological mediators between childhood adversities/trauma and suicidal behavior.

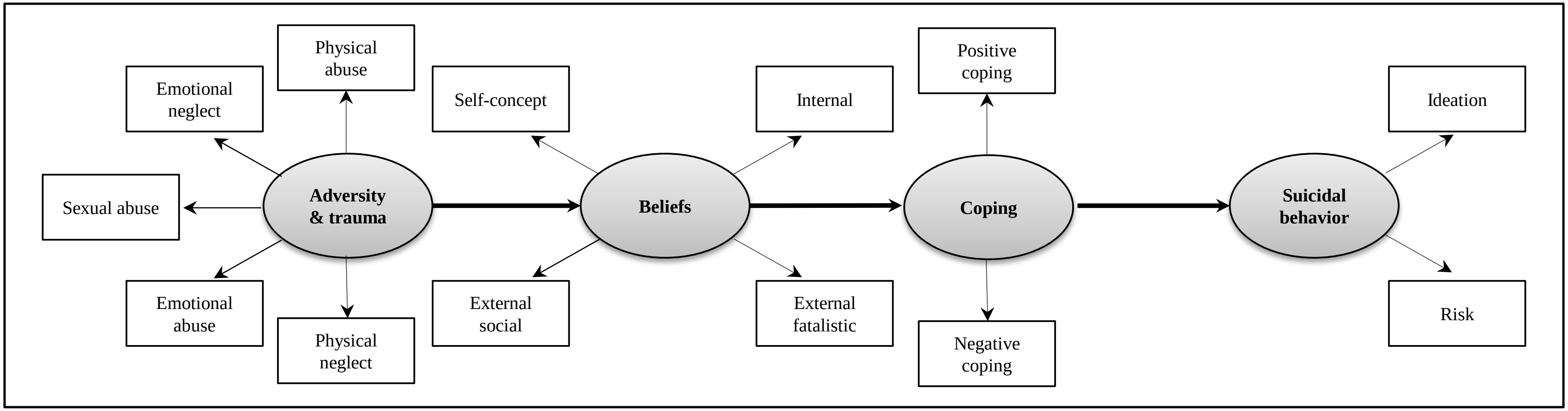

Figure S2b. Psychological mediators between childhood adversities/trauma and suicidal behavior.

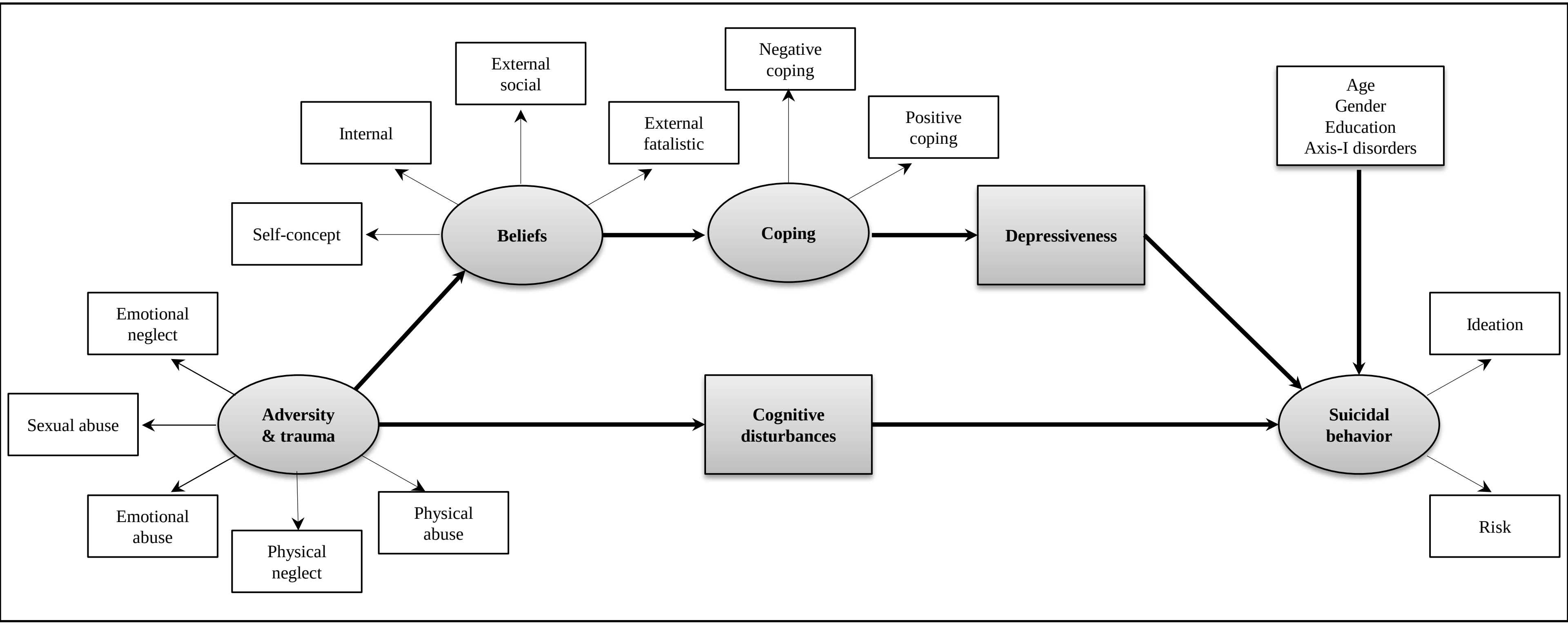

Figure S3a. Psychological and symptomatic mediators between childhood adversities/trauma and suicidal behavior.

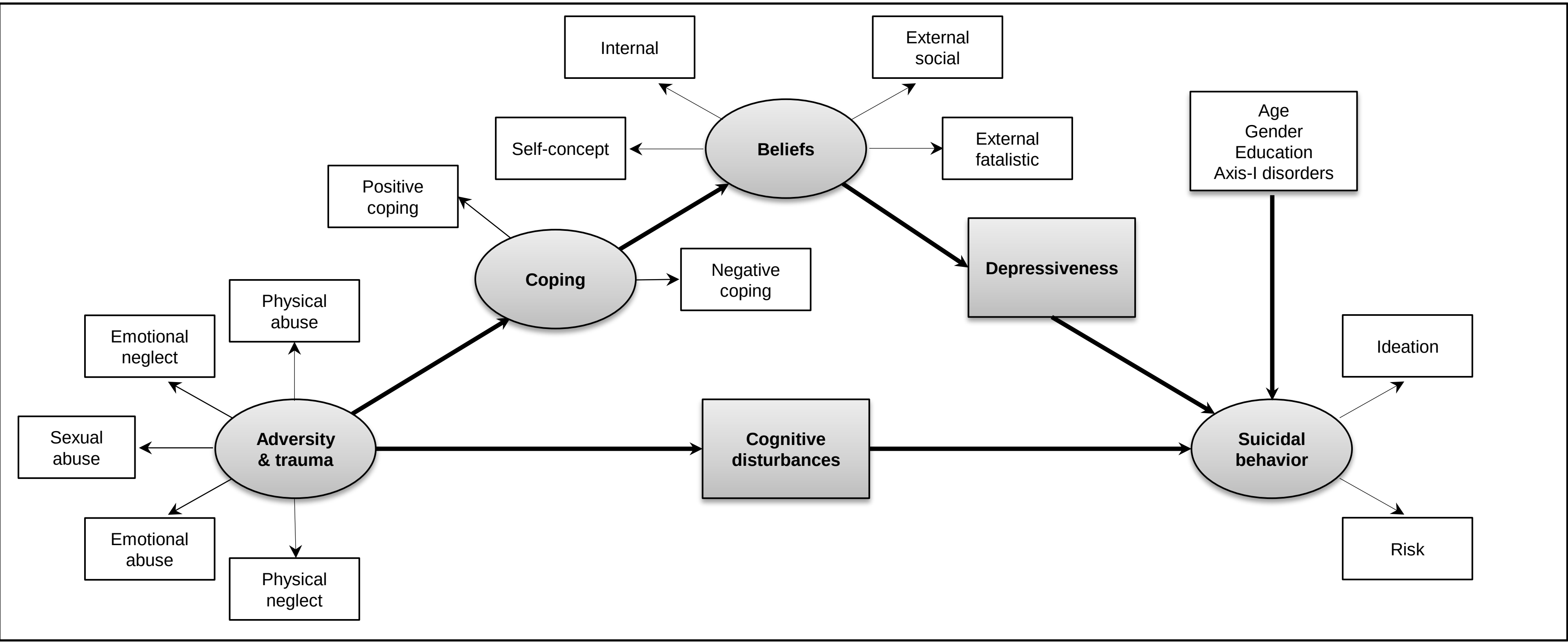

Figure S3b. Psychological and symptomatic mediators between childhood adversities/trauma and suicidal behavior.

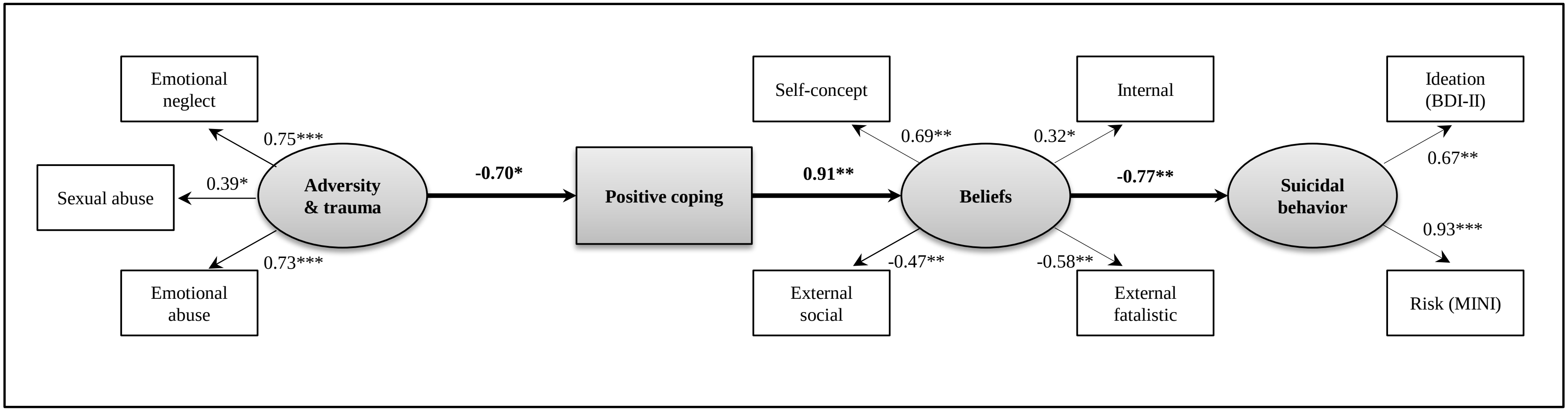

Figure S4. Psychological mediators between childhood adversities/trauma and suicidal behavior.
Model fit indices: $\chi^2_{(33)}$=43.25, *P*=0.109; CFI=0.90; RMSEA=0.07, *P*=0.305, WRMR=0.60
Standardized indirect effect, IE=0.52; 95% CIs=0.15, 0.90; *P*=0.007
Note. Rectangles present observed manifest variables, ovals unobserved latent variables; values are standardized path coefficients.
**P<0.05,* ***P*<.01, ****P*<.001

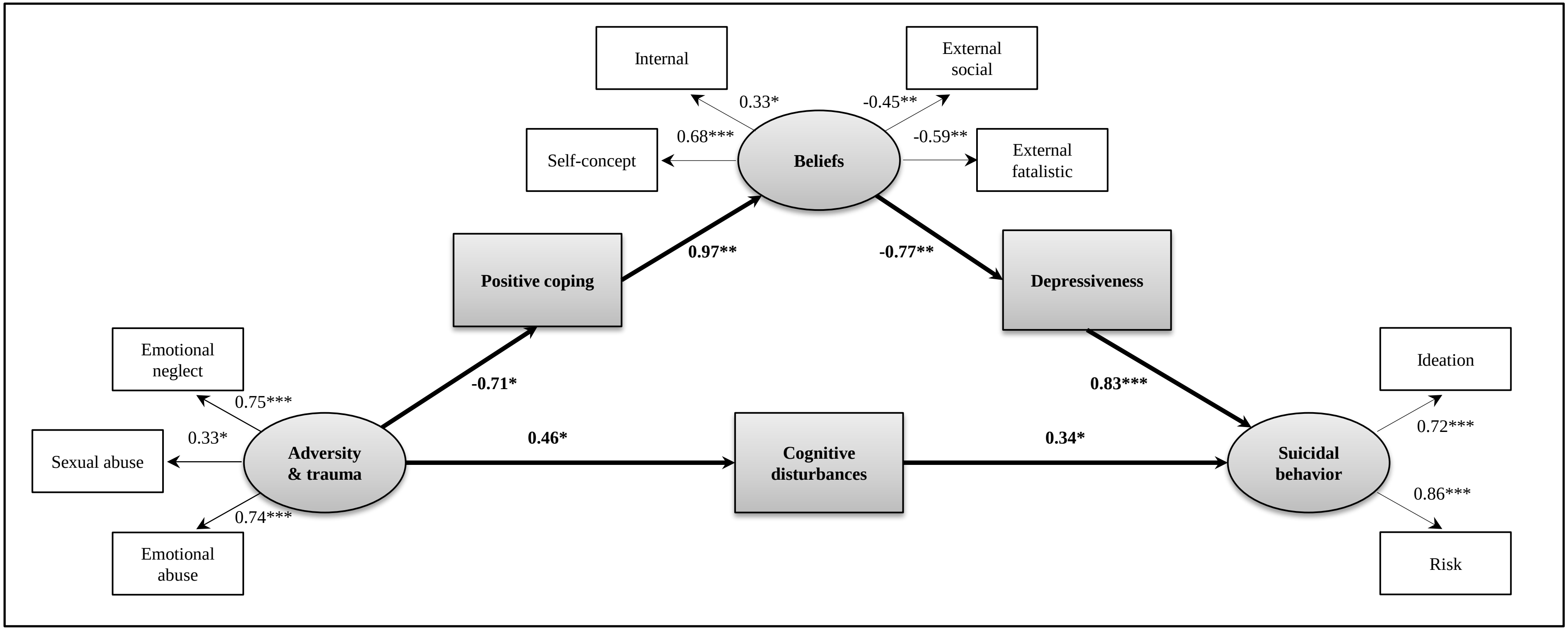

Figure S5. Psychological and symptomatic mediators between childhood adversities/trauma and suicidal behavior.
Model fit indices: $\chi^2_{(51)}$=59.86, *P*=0.190; CFI=0.95; RMSEA=0.049, *P*=0.489; WRMR=0.59
Standardized indirect effect through coping - beliefs - depressiveness: IE=0.44; 95% CIs=0.10, 0.78; *P*<0.001
and through cognitive disturbances: IE=0.16, 95% CIs=0.01, 0.31; *P*=0.049
Note. Rectangles present observed manifest variables, ovals unobserved latent variables; values are standardized path coefficients.
**P<0.05*, ***P*<.01, ****P*<.001
